# Two new species of the primitively segmented spider genus *Songthela* from Hunan Province, China (Mesothelae, Liphistiidae)

**DOI:** 10.3897/zookeys.937.50548

**Published:** 2020-06-01

**Authors:** Dengqing Li, Fengxiang Liu, Daiqin Li, Xin Xu

**Affiliations:** 1 College of Life Sciences, Hunan Normal University, Changsha, Hunan 410081, China Hunan Normal University Changsha China; 2 State Key Laboratory of Biocatalysis and Enzyme Engineering, and Centre for Behavioural Ecology and Evolution (CBEE), School of Life Sciences, Hubei University, 368 Youyi Road, Wuhan 430062, Hubei Province, China Hubei University Hubei China; 3 Department of Biological Sciences, National University of Singapore, 14 Science Drive 4, Singapore 117543, Singapore National University of Singapore Singapore Singapore

**Keywords:** Araneae, COI, morphology, *
Songthela
*, taxonomy

## Abstract

This study reports two new species of the primitively segmented spider genus *Songthela* from Hunan Province, China, based on morphological characters: *S. huangyang***sp. nov.** (♂♀), *S. xiangnan***sp. nov.** (♂♀). Additional material also facilitates a more accurate description of *S. goulouensis* (Yin, 2001) with the first description of the male. Nucleotide data for the barcoding gene, cytochrome c oxidase subunit I (COI), is also provided for these three species.

## Introduction

In spite of being a species-poor lineage, the primitively segmented spider family Liphistiidae has recently received much attention (e.g., Xu et al. 2015a, b, 2016, 2017a, 2019; Schwendinger 2017; Aung et al. 2019; Schwendinger et al. 2019) because of its pivotal position in the spider tree of life (Platnick and Gertsch 1976; Xu et al. 2015b). As the sole sister to all other extant spiders, Liphistiidae bears unique plesiomorphies such as the presence of abdominal tergites and the spinnerets located ventrally, in the middle of the abdomen (Bristowe 1975; Haupt 2003; Schwendinger and Ono 2011; Xu et al. 2015a, b, 2016, 2019). Extant liphistiids are confined to East and Southeast Asia where most species are highly endemic (Bristowe 1975; Haupt 2003; Xu et al. 2017a, b; World Spider Catalog 2020). Liphistiidae currently contains 135 species in eight genera (World Spider Catalog 2020). *Heptathela* Kishida, 1923 (Xu et al. 2019), *Ganthela* Xu & Kuntner, 2015 (Xu et al. 2015c) and *Ryuthela* Haupt, 1982 (Xu et al. 2017a) have been comprehensively reviewed, but no revision has yet been performed on the remaining five genera. During our review of the genus *Songthela* Ono, 2000 from China, we discovered several new taxa in Hunan Province.

The genus *Songthela* was erected based on the type species *S. hangzhouensis* (Chen, Zhang & Zhu, 1981) using female morphology by Ono (2000), but considered as a synonym of *Sinothela* Haupt, 2003 by Haupt (2003) based on male palp morphology. In 2011, Schwendinger and Ono synonymized *Songthela* with *Heptathela* due to the lack of diagnostic characters (Schwendinger and Ono 2011). However, Xu and colleagues recently recovered *Songthela* by integrating molecular data with morphological characters (Xu et al. 2015a). Currently, *Songthela* consists of 14 described species, mostly from southern China, but also including *S. sapana* (Ono, 2010) from northern Vietnam (World Spider Catalog 2020).

**Figure 1.**
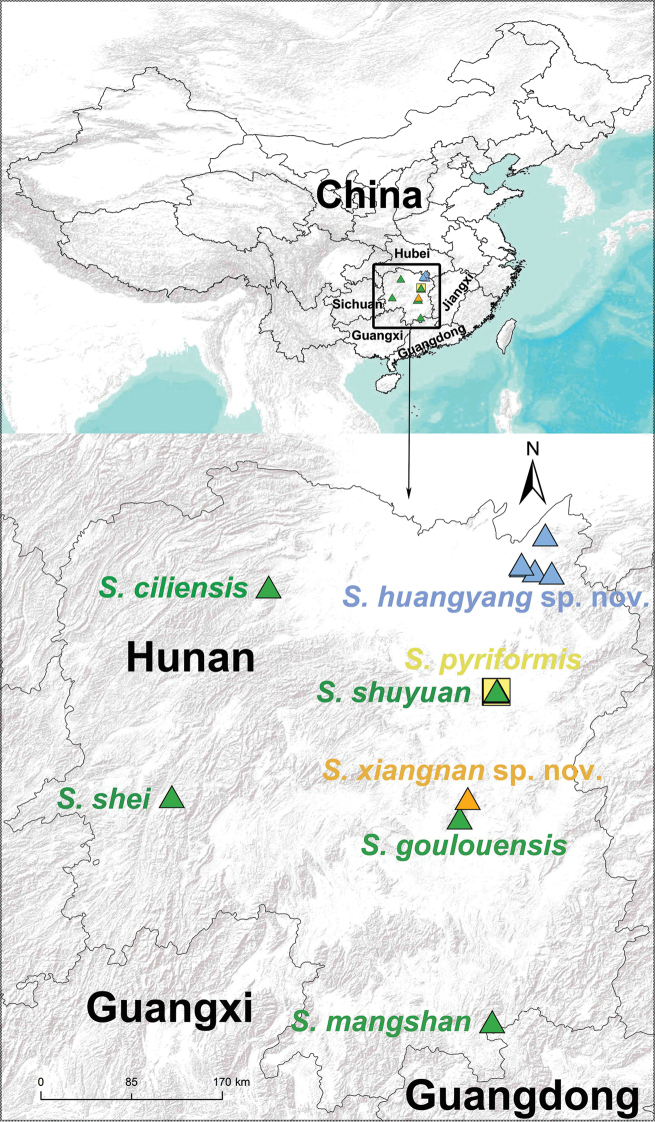
Map showing the localities of eight *Songthela* species that currently are distributed in Hunan Province, China. The distributions of two new species are indicated in blue and orange solid triangles, and the type localities of six known species are indicated in green solid triangles as well as yellow solid square.

Until now, six *Songthela* species have been reported from Hunan Province: *S. ciliensis* (Yin, Tang & Xu, 2003), *S. goulouensis* (Yin, 2001), *S. mangshan* (Bao, Yin & Xu, 2003), *S. pyriformis* Li, Liu & Xu, 2019, *S. shei* (Xu & Yin, 2001), and *S. shuyuan* Li, Liu & Xu, 2019 (Fig. 1). In this study, we describe two new *Songthela* species collected in Hunan Province based on male and female genital morphology. We also provide the COI GenBank accession numbers of the holotypes of the two new species for future identification. In addition, the species *S. goulouensis* was firstly diagnosed and described based on female morphology, and one specimen was found to bear two sets of female genitalia, named as “didymous phenomenon” (Yin 2001). However, Schwendinger and Ono (2011) attributed the “didymous phenomenon” to the duality of receptacular clusters. The same phenomenon was also reported in the purseweb spider family Atypidae (Li et al. 2018). To resolve this issue, we examined the types of *S. goulouensis* and provide its redescription.

## Material and methods

All specimens were collected from Hunan Province, China (Fig. 1). Subadult males/females were brought back to the laboratory and reared to adulthood. The right four legs of adults were removed, preserved in 100% ethanol, and stored at –80 °C for molecular work, and the remains were kept in 80% ethanol for morphological examination. The specimens were photographed using a digital camera MC170 HD mounted on Leica M205C stereomicroscope. The soft tissues of female genitalia were dissolved using 10 mg/ml trypsase (Bomei Biotech Company, Hefei, Anhui, China) for at least three hours at the room temperature. Male and female genitalia were then photographed using the digital camera CCD mounted on an Olympus BX53 compound microscope. All the holotypes and voucher specimens were deposited at the College of Life Sciences, Hunan Normal University (HNU), Changsha, China.

Four female types of *S. goulouensis* deposited in Hunan Normal University were also examined. All the vouchers lacked the specimen codes, which were also not indicated in the original description of Yin (2001). To ease the description, each type specimen was assigned a code, the lectotype was designated, *S. goulouensis* was then redescribed using the newly collected male and female specimens at the same locality, and these types were also photographed for comparison. The COI GenBank accession number of this species using these specimens is also provided.

All measurements are given in millimeters, leg and palp measurements are given in the following order: total leg length (femur + patella + tibia + metatarsus + tarsus), total palp length (femur + patella + tibia + tarsus).

Abbreviations used:

**ALE** anterior lateral eyes;

**AME** anterior median eyes;

**BL** body length;

**CL** carapace length;

**Co** conductor;

**CT** contrategulum;

**CW** carapace width;

**E** embolus;

**OL** opisthosoma length;

**OW** opisthosoma width;

**PC** paracymbium;

**PLE** posterior lateral eyes;

**PME** posterior median eyes;

**RC** receptacular cluster;

**T** tegulum.

The total genomic DNA was extracted from spider legs using the Animal Genomic DNA Isolation Kit (Kangwei Biotech, Beijing, China). The primer pair LCO1490/HCO2198 (Folmer et al. 1994) was used for amplification of COI. The PCR reaction protocol was initial denaturation at 95 °C for 5 min; 35 cycles of denaturation at 95 °C for 1 min, annealing at 40 °C for 1 min, and elongation at 72 °C for 30s; and final extension at 72 °C for 7 min (Xu et al. 2015c). The PCR reactions had a total volume of 25 µl, consisting of 12.5 µl of 2×Taq or 2×Es MasterMix (KangWei Biotech, Beijing, China), 1 µl of each forward and reverse 10 µM primer, 1 µl of genomic DNA, and 9.5 µl of double-distilled H_2_O. The PCR products were visualized by agarose gel electrophoresis (1% agarose). PCR products were purified and sequenced at Tsingke Biotechnology Company (Changsha, China).

## Taxonomy

### Genus Songthela

Taxon classificationAnimaliaAraneaeLiphistiidae

Ono, 2000

41D20A00-B76B-5FCD-A90C-5280C7BBFCBB

#### Type species.

*Heptathela hangzhouensis* Chen, Zhang & Zhu, 1981.

#### Diagnosis.

*Songthela* males can be distinguished from those of all other heptatheline genera by the conductor with the proximal portion relatively narrow, the distal portion with one or two apical spines (Figs 3B, E, 5B, E, 7B, E); by the distal of the embolus slightly sclerotized, with a wide and flat opening (Figs 3C, D, 5A, D, 7A, D); and by the contrategulum with a serrate margin (Figs 3C, D, 5A, D, G, 7A, D, G). *Songthela* females differ from those of all other heptatheline genera by four receptacular clusters with smooth surface, the median ones with relatively long sturdy genital stalks, four receptacular clusters along the anterior margin of the bursa copulatrix or the lateral ones situated dorsolaterally (Figs 3H–J, 5H, 8A–D, 8I–L, 9A–D).

#### Distribution.

China and northern Vietnam.

### Songthela huangyang sp. nov.

Taxon classificationAnimaliaAraneaeLiphistiidae

5F3B2167-8C42-59C3-A071-0BE610723479

http://zoobank.org/DA8D876A-C5CA-44A3-A206-0DA36251D59D

Figures 2 , 3

#### Type material.

***Holotype***: China·1 ♂; Hunan Province, Yueyang City, Huangyang Group; 29.26°N, 113.15°E; alt. 85 m; 26 Jun. 2018; D. Li, F.X. Liu, X. Xu, D.Q. Li leg.; XUX–2018–081 (matured on 22 September 2018 at HNU).

***Paratypes***: China·1 ♂, 2 ♀♀; same data as for holotype; XUX–2018–080, 082, XUX–2018–083 (matured on 20 October 2018 at HNU)·1 ♂; Hunan Province, Yueyang City, Linxiang City, Xiacaojiachong Village; 29.51°N, 113.35°E; alt. 89 m; 24 Jun. 2018; D. Li, F.X. Liu, X. Xu, D.Q. Li leg.; XUX–2018–056 (matured on 20 October 2018 at HNU)·1 ♀; Hunan Province, Yueyang City, Yueyang County, Gangkou Town, Gangkou Village; 29.18°N, 113.40°E; alt. 21 m; 25 Jun. 2018; D. Li, F.X. Liu, X. Xu, D.Q. Li leg.; XUX–2018–059·1 ♂; Hunan Province, Yueyang City, Yueyang County, Gangkou Town, Yishan Village; 29.20°N, 113.26°E; alt. 55 m; 25 Jun. 2018; D. Li, F.X. Liu, X. Xu, D.Q. Li leg.; XUX–2018–077 (matured on 20 October 2018 at HNU).

#### Diagnosis.

Males of *S. huangyang* sp. nov. resemble those of *S. ciliensis*, *S. mangshan* and *S. pyriformis* by the conductor with one apical spine, but can be distinguished from *S. ciliensis* and *S. mangshan* by the narrow base of the conductor (Fig. 3B, E); from *S. pyriformis* by the thinner conductor apical spine, the contrategulum with the densely denticulate margin, and the narrower tegulum (Fig. 3D–G); from the other *Songthela* species by the conductor base narrow with one apical spine (Fig. 3B, E); and by the narrower tegulum (Fig. 3F). Females of *S. huangyang* sp. nov. differ from those of other *Songthela* species by four receptacular clusters along the anterior margin of the bursa copulatrix, and the median pairs with obscure slender genital stalks (Fig. 3I, J, L, M).

#### Description.

**Figure 2.**
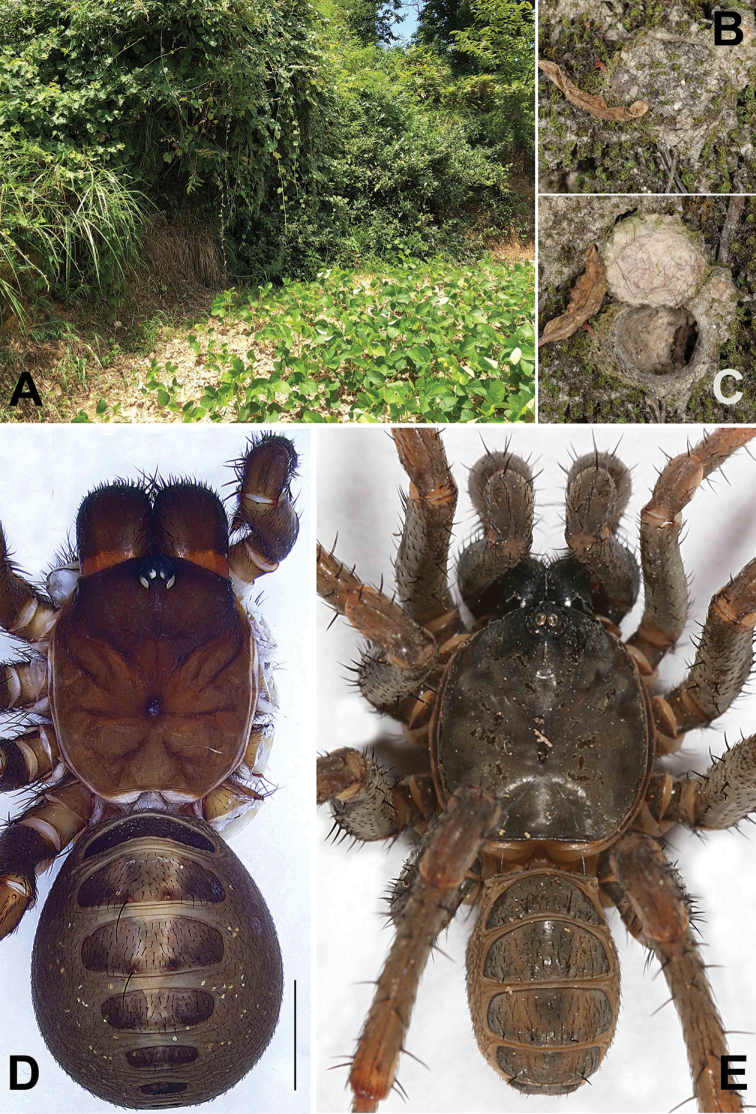
Microhabitat and general somatic morphology of *Songthela huangyang* sp. nov. (XUX–2018–082, female; XUX–2018–083, male) **A** microhabitat **B, C** the trapdoor with the door closed and open **D, E** dorsal view. Scale bar: 3 mm (**D**).

**Male** (holotype; Fig. 2E). Carapace black brown and opisthosoma light brown in alcohol; fourth to sixth tergite yellow brown, remaining tergites dark brown; sternum narrow, much longer than wide; few fine pointed hairs running over ocular area; chelicerae robust with promargin of cheliceral groove with 12 denticles of variable size; legs with sturdy hairs and spines; opisthosoma with 12 tergites, the second to fifth tergite larger than remaining ones and fourth the largest; eight spinnerets. Measurements: BL 9.29, CL 4.33, CW 3.94, OL 4.73, OW 2.79; ALE > PLE > PME > AME; leg I 14.73 (4.09 + 1.86 + 2.94 + 3.79 + 2.05), leg II 15.03 (4.10 + 1.81 + 2.83 + 4.02 + 2.27), leg III 16.36 (4.21 + 1.84 + 2.96 + 4.73 + 2.62), leg IV 21.05 (5.14 + 2.05 + 3.97 + 6.45 + 3.44).

**Figure 3.**
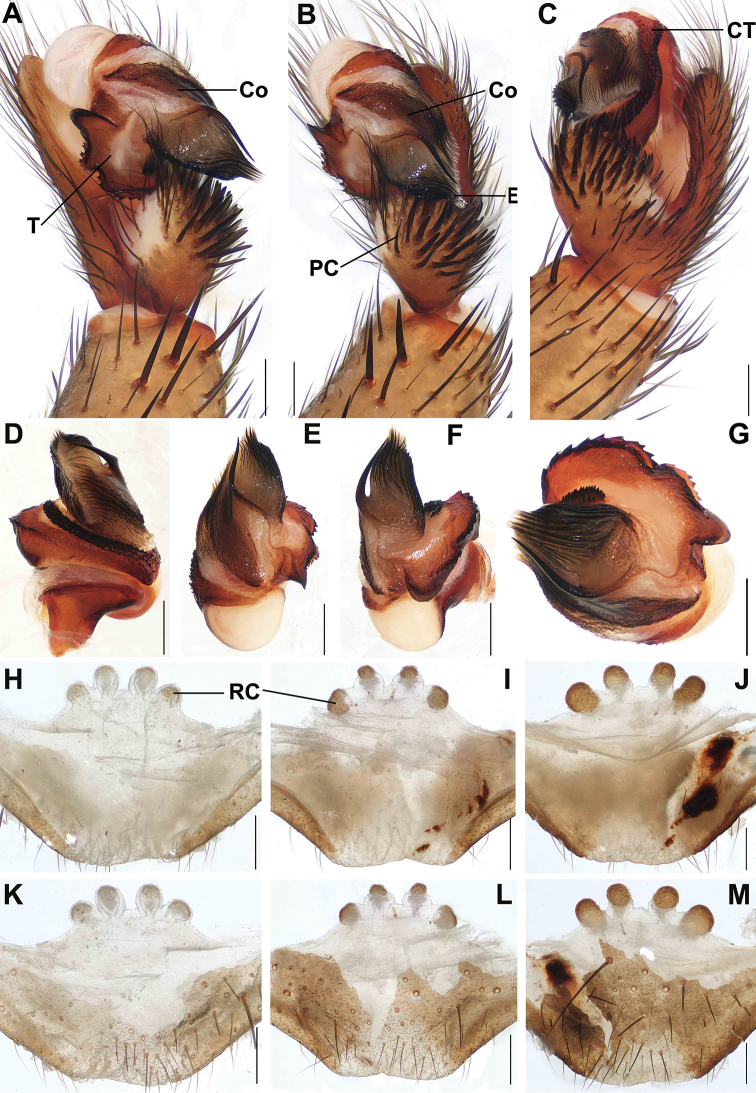
Male and female genital anatomy of *Songthela huangyang* sp. nov. **A, D** palp prolateral view **B, E** palp ventral view **C, F** palp retrolateral view **G** palp distal view **H–J** vulva dorsal view **K–M** vulva ventral view **A–C** XUX–2018–081 (holotype) **D–G** XUX–2018–083 **H, K** XUX–2018–059 **I, L** XUX–2018–080 **J, M** XUX–2018–082. Scale bars: 0.3 mm (**A–M**).

***Palp*.** Paracymbium with numerous setae and spines on the tip (Fig. 3A–C); contrategulum with a densely denticulate margin (Fig. 3D); tegulum narrow with a serrate marginal apophysis and a finger-like terminal apophysis (Fig. 3F, G); conductor situated ventro-proximally from the embolus, its base narrow and fused with embolus, distal portion free, and sharply narrowing to a apical spine (Fig. 3A, B, E); embolus with a wide and flat opening (Fig. 3C, D).

**Female** (XUX–2018–082; Fig. 2D). Carapace red brown and opisthosoma brown in alcohol; fourth to sixth tergite grey and brown, remaining tergites dark brown; sternum narrow, much longer than wide; few fine pointed hairs running over ocular area; chelicerae robust with promargin of cheliceral groove with eleven denticles; legs with sturdy hairs and spines; opisthosoma with 12 tergites, the second to fifth tergite larger than remaining ones and fourth the largest; eight spinnerets. Measurements: BL 14.15, CL 5.97, CW 5.46, OL 7.73, OW 6.55; ALE > PLE > PME > AME; palp 11.05 (3.64 + 2.07 + 2.47 + 2.87), leg I 12.80 (3.95 + 2.33 + 2.47 + 2.61 + 1.44), leg II 12.40 (3.67 + 2.26 + 2.28 + 2.66 + 1.53), leg III 12.91 (3.53 + 2.31 + 2.26 + 3.13 + 1.68), leg IV 18.24 (4.76 + 2.67 + 3.31 + 5.03 + 2.47).

***Female genitalia*.** Four receptacular clusters along the anterior margin of the bursa copulatrix, the median pair with obscure slender genital stalks, smaller than or similar to the lateral ones; the lateral receptacular clusters without genital stalks (Fig. 3H–M); the posterior part of the genital area inverted trapezoid (Fig. 3H–M).

#### Variation.

Males and females vary in body size. The range of measurements in males as follows (*N* = 4): BL 9.29–10.78, CL 4.33–5.07, CW 3.94–5.02, OL 4.43–4.73, OW 2.77–3.11; females (*N* = 3): BL 9.23–14.15, CL 3.55–5.97, CW 4.01–5.46, OL 5.36–7.73, OW 4.44–6.55. In addition, female genitalia show slight intraspecific variation: the genital stalks of the median pair are slender (Fig. 3I, J, L, M), or four receptacular clusters are slightly sclerotized, perhaps because of the early stage of maturity of the specimen (Fig. 3H, K); or the genital stalks of the median pair have the obscure ribbed distal portion (Fig. 3I, L).

#### Etymology.

The species epithet, a noun in apposition, refers to the type locality.

#### Distribution.

Hunan (Yueyang) Province, China.

#### GenBank accession number.

Holotype (XUX–2018–081): MT102213.

### Songthela xiangnan sp. nov.

Taxon classificationAnimaliaAraneaeLiphistiidae

BD560AAC-2C6B-56AE-B59C-35C9BCC645CD

http://zoobank.org/C1546A27-6E26-4271-8457-91CA59E072A9

Figures 4 , 5

#### Type material.

***Holotype***: China·1 ♂; Hunan Province, Hengyang City, Mt. Heng, Xiangnan Temple; 27.28°N, 112.69°E; alt. 959 m; 18 Aug. 2011; F.X. Liu, X. Xu, R. Xiao leg.; XUX–2011–063.

***Paratypes***: China·2 ♀♀; same data as for holotype; XUX–2011–070, 071.

#### Diagnosis.

Male of *S. xiangnan* sp. nov. resembles that of *S. pluma*, but can be distinguished from the latter by the conductor with two apical spines, the longer spine with a bifid apex (Fig. 5A, B, D, E, G), by the contrategulum with only one dentate margin and with a small apophysis located proximally (Fig. 5A, B, D, E), and by the tegulum with a finger-like terminal apophysis in retrolateral view (Fig. 5C, F); from that of other *Songthela* species by the central part of the conductor with several short spines, the blade-shaped conductor spine with a bifid apex (Fig. 5A, B, D, E, G). Females of *S. xiangnan* sp. nov. differ from those of *S. pluma* by the longer genital stalks of the median receptacular clusters (Fig. 5H), and the smaller lateral receptacular clusters with obscure genital stalks (Fig. 5H); from those of other *Songthela* species by the median receptacular clusters with longer and trachea-shaped genital stalks, obviously larger than the lateral ones, the lateral receptacular clusters with obscure genital stalks, situated slightly dorsolaterally (Fig. 5H).

#### Description.

**Figure 4.**
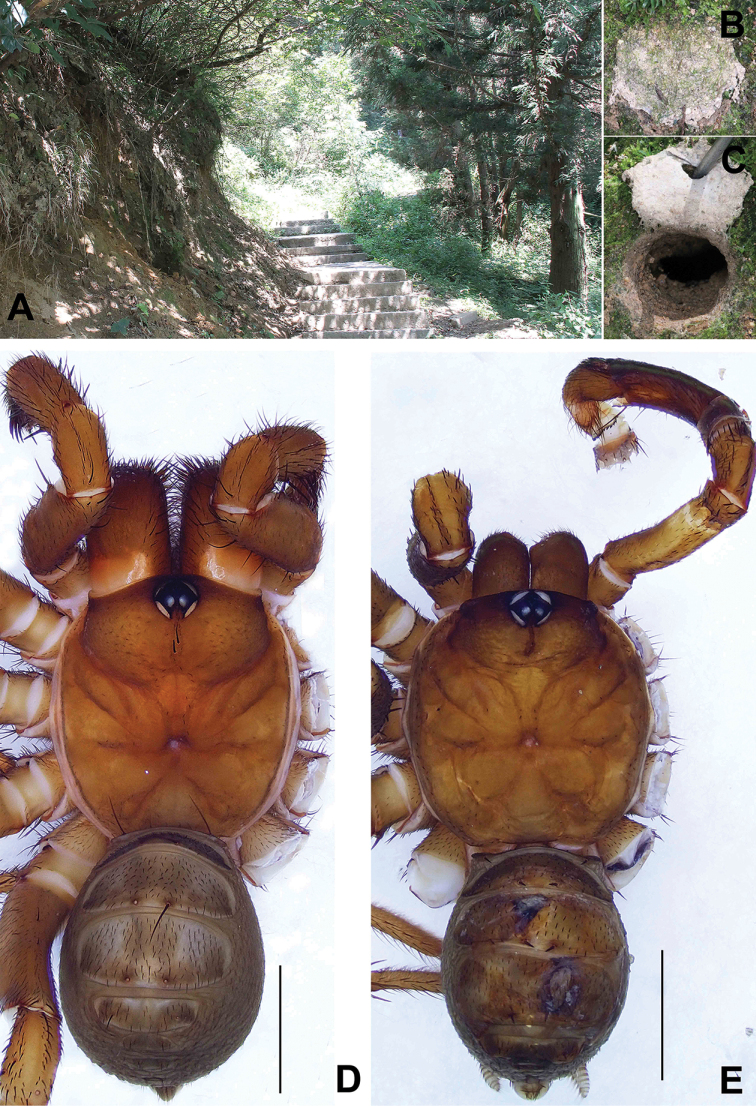
Microhabitat and general somatic morphology of *Songthela xiangnan* sp. nov. (XUX–2011–071, female; XUX–2011–063, male) **A** microhabitat **B, C** the trapdoor with the door closed and open **D, E** dorsal view. Scale bars: 3 mm (**D, E**).

**Male** (holotype; Fig. 4E). Carapace yellow with several short hairs on the margin; opisthosoma light brown; tergites yellowish-brown; sternum narrow; few fine pointed hairs running over ocular area; chelicerae robust with promargin of cheliceral groove with 12 denticles; legs with sturdy hairs and spines; opisthosoma with 12 tergites, the second to fifth tergite larger than remaining ones and third the largest; six spinnerets. Measurements: BL 10.80, CL 5.51, CW 5.48, OL 5.18, OW 4.10; ALE > PLE > PME > AME; leg I 20.24 (5.77 + 2.43 + 4.41 + 5.34 + 2.29), leg II 20.51 (5.63 + 2.43 + 4.28 + 5.69 + 2.48), leg III 21.83 (5.45 + 2.49 + 4.32 + 6.71 + 2.86), leg IV 28.2 (7.15 + 2.74 + 5.79 + 8.97 + 3.55).

**Figure 5.**
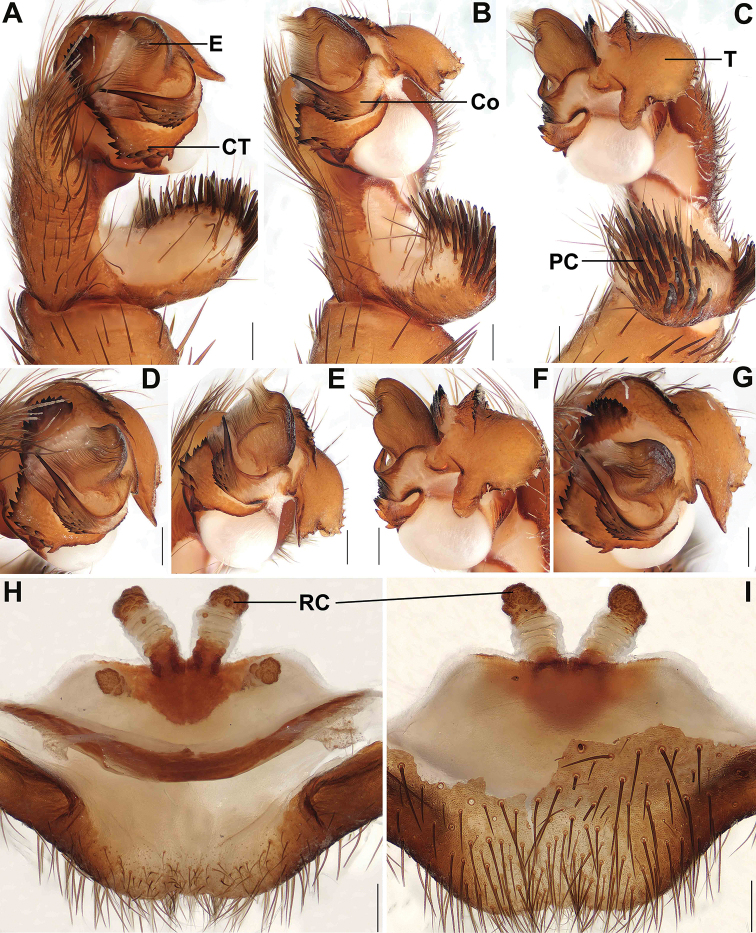
Male and female genital anatomy of *Songthela xiangnan* sp. nov. **A, D** palp prolateral view **B, E** palp ventral view **C, F** palp retrolateral view **G** palp distal view **H** vulva dorsal view **I** vulva ventral view **A–G** XUX–2011–063 (holotype) **H–I** XUX–2011–070. Scale bars: 0.3 mm (**A–I**).

***Palp*.** Prolateral side of paracymbium unpigmented and unsclerotized, with numerous setae and spines on the tip (Fig. 5A); contrategulum with serrate margin and with a small apophysis located proximally (Fig. 5A, B, D, E); tegulum with a dentate marginal apophysis and the dorsal extension of terminal apophysis, and a long finger-like terminal apophysis in retrolateral view (Fig. 5C, F); conductor base wide and fused with embolus, unsmooth middle portion covered with several short spines, a long blade-shaped spine with a bifid apex and a short spine basally (Fig. 5A, B, D, E); embolus with a flat opening in distal portion and ridged apophysis in middle ventral portion (Fig. 5A–G).

**Female** (XUX–2011–070; Fig. 4D). Carapace yellow brown with several hairs on the margin; opisthosoma brown; fourth to sixth tergite light brown with some brown flecks, remaining tergites dark brown; sternum narrow; few fine pointed hairs running over ocular area; chelicerae robust with promargin of cheliceral groove with 12 denticles; legs with sturdy hairs and spines; opisthosoma with 12 tergites, the second to fifth tergite larger than remaining ones and fourth the largest; six spinnerets. Measurements: BL 14.31, CL 6.44, CW 5.94, OL 7.04, OW 6.18; ALE > PLE > PME > AME; palp 12.59 (4.35 + 2.31 + 2.64 + 3.29), leg I 14.28 (4.64 + 2.52 + 2.78 + 2.80 + 1.54), leg II 13.76 (4.25 + 2.42 + 2.52 + 3.00 + 1.57), leg III 14.32 (4.08 + 2.44 + 2.53 + 3.41 + 1.86), leg IV 20.37 (5.72 + 2.94 + 3.74 + 5.30 + 2.67).

***Female genitalia*.** The median pair of the receptacular clusters along the anterior margin of the bursa copulatrix, with long, trachea-shaped and slightly ventral-tilted genital stalks close to each other basally but separated from each other distally; the middle receptacular clusters obviously larger than the lateral ones; the lateral receptacular clusters with obscure genital stalks, situated slightly dorsolaterally (Fig. 5H, I); the anterior margin of the bursa copulatrix trapezoid (Fig. 5H, I).

#### Variation.

The range of measurements in females as follows (*N* = 2): BL 12.56–14.31, CL 6.26–6.44, CW 5.67–5.94, OL 5.78–7.04, OW 4.65–6.18.

#### Etymology.

The species epithet, a noun in apposition, refers to the type locality.

#### Distribution.

Hunan (Hengyang) Province, China.

#### GenBank accession number.

**Holotype**: XUX–2011–063: MT102212.

### Songthela goulouensis

Taxon classificationAnimaliaAraneaeLiphistiidae

(Yin, 2001)

7A9D8F0E-4C7E-52BB-AFDC-A2C3F6E17C32

**Figure 6.**
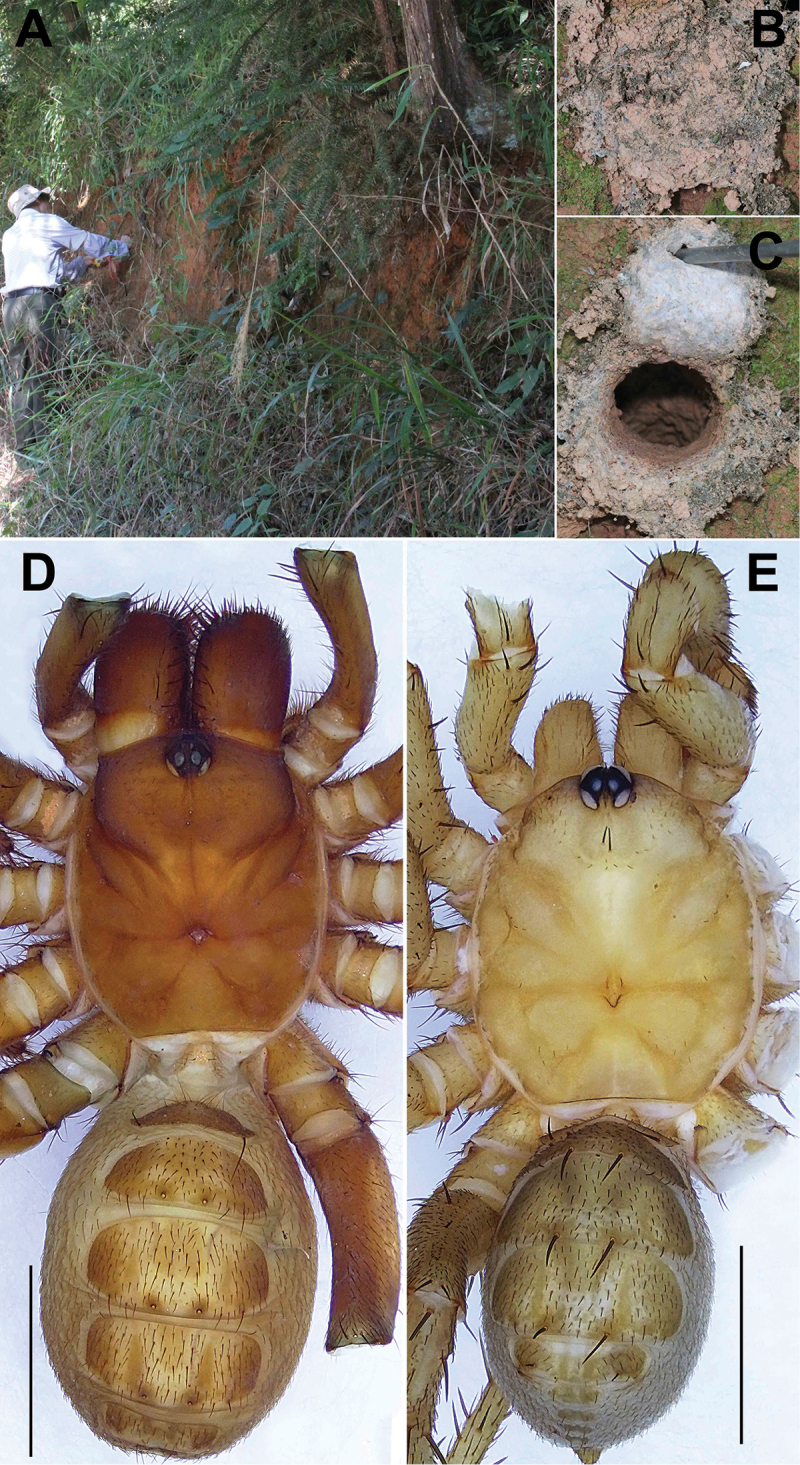
Microhabitat and general somatic morphology of *Songthela goulouensis* (GL–1997–001, female; XUX–2011–110A, male) **A** microhabitat **B, C** the trapdoor with the door closed and open **D, E** dorsal view. Scale bars: 3 mm (**D, E**).

**Figure 7.**
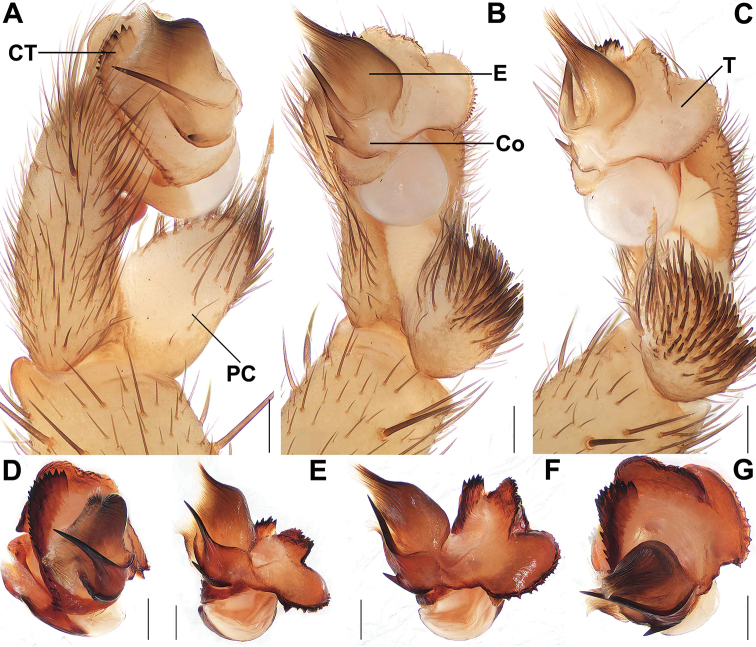
Male genital anatomy of *Songthela goulouensis***A, D** palp prolateral view **B, E** palp ventral view **C, F** palp retrolateral view **G** palp distal view **A–C** XUX–2011–110A **D–G** XUX–2011–098. Scale bars: 0.3 mm (**A–G**).

**Figure 8.**
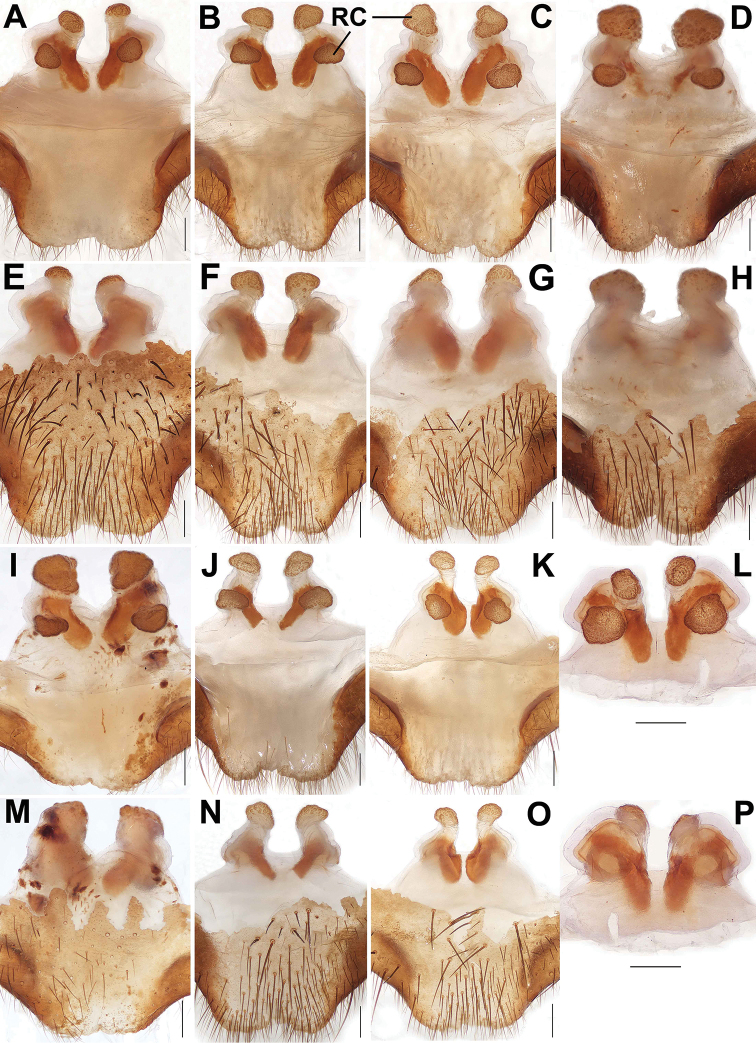
Female genital anatomy of *Songthela goulouensis***A–D, I–L** vulva dorsal view **E–H, M–P** vulva ventral view **A, E** XUX–2011–093 **B, F** XUX–2011–095 **C, G** XUX–2011–099 **D, H** XUX–2011–114 **I, M** XUX–2011–104 **J, N** XUX–2011–115 **K, O** XUX–2011–106 **L, P** XUX–2011–109. Scale bars: 0.3 mm (**A–P**).

**Figure 9.**
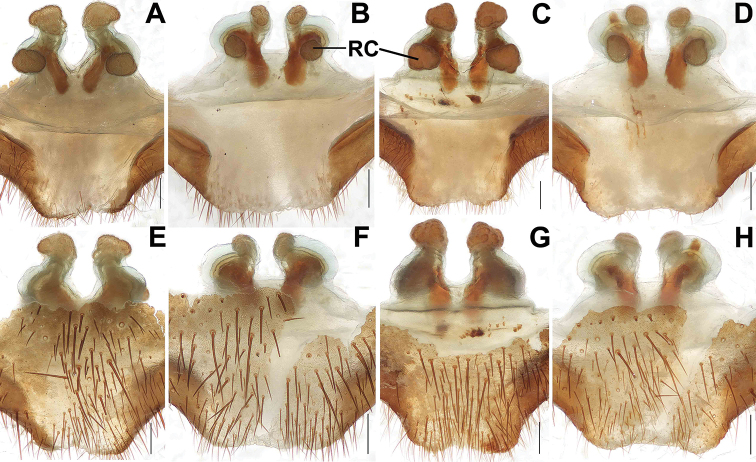
Female genital anatomy of *Songthela goulouensis***A–D** vulva dorsal view **E–H** vulva ventral view **A, E** GL–1997–002 **B, F** GL–1997–001 (lectotype) **C, G** GL–1997–003 **D, H** GL–1997–004. Scale bars: 0.3 mm (**A–H**).

Figures 6 , 7 , 8 , 9

Heptathela goulouensis Yin, 2001: 297 (♀♀, from Gouloufeng, Hengyang City, Hunan Province, China, collected by X.J. Peng on 1 August 1997; deposited in HNU, examined); Yin 2001: 2; Yin et al. 2012: 114;
Songthela goulouensis Xu et al., 2015: 141.

#### Material examined.

China·2 ♂♂, 11 ♀♀; Hunan Province, Hengyang City, Gouloufeng, nearby Yuwang Hotel; 27.12°N, 112.63°E; alt. 609–637 m; 20 Aug. 2011; F.X. Liu, X. Xu, R. Xiao leg.; XUX–2011–093, 095, 096, 098, 099, 100, 104, 105 106, 108, 109, 110, 110A·5 ♀♀; Hunan Province, Hengyang City, Gouloufeng; 27.12°N, 112.62°E; alt. 556–558 m; 20 Aug. 2011; F.X. Liu, X. Xu, R. Xiao leg.; XUX–2011–111, 113 to 116; 4 ♀♀; Hunan Province, Hengyang City, Gouloufeng; alt. 1500 m; 1 Aug. 1997; X.J. Peng leg.; GL–1997–001 (lectotype) to 004.

#### Diagnosis.

Males of *S. goulouensis* resemble those of *S. hangzhouensis* by the conductor with two spines, but can be distinguished from the latter by the shorter spine located near the conductor margin, the longer spine extended over the embolus opening margin (Fig. 7A, B, D, E), and by the tegulum with a smaller terminal apophysis in retrolateral view (Fig. 7C, F); from *S. shuyuan* by the conductor with a narrower base and a longer apical spine (Fig. 7B, E), by the embolus with a slightly curved distal margin (Fig. 7A–E), and by the tegulum with a smaller terminal apophysis in retrolateral view (Fig. 7C, F); from those of the other *Songthela* species by the smooth conductor with two apical spines (Fig. 7A–G). Females of *S. goulouensis* differ from those of the other *Songthela* species by the median receptacular clusters located at the two peaks of the anterior margin of the bursa copulatrix, with obvious genital stalks, the lateral ones situated dorsolaterally, close to the base of the middle genital stalks, with obscure genital stalks (Figs 8A–D, 8I–L, 9A–D).

#### Description.

**Male** (XUX–2011–110A; Fig. 6E). Carapace light yellow with several hairs on the margin; opisthosoma light brown; fourth to sixth tergite brown with some light brown flecks, remaining tergites brown; sternum narrow; few fine pointed hairs running over ocular area; chelicerae robust with promargin of cheliceral groove with eleven denticles; legs with sturdy hairs and spines; opisthosoma with 12 tergites, the second to fifth tergite larger than remaining ones and fourth the largest; seven spinnerets. Measurements: BL 9.67, CL 4.58, CW 4.33, OL 4.73, OW 3.33; ALE > PLE > PME > AME; leg I 14.53 (4.03 + 1.91 + 2.93 + 3.60 + 2.06), leg II 14.81 (3.75 + 1.86 + 2.89 + 3.97 + 2.34), leg III 16.25 (3.80 + 1.87 + 2.94 + 4.81 + 2.83), leg IV 21.50 (4.72 + 2.24 + 4.10 + 6.87 + 3.57).

***Palp*.** Paracymbium unpigmented and unsclerotized in prolateral view, with numerous setae and spines on the tip (Fig. 7A); contrategulum with serrate margin (Fig. 7A, D, G); tegulum with a dentate marginal apophysis and the dorsal extension of terminal apophysis, and with a small terminal apophysis in retrolateral view (Fig. 7C, F); the smooth conductor base fused with embolus, with two free apical spines, the short one located at the one third of the conductor and close to the conductor margin, the long one extended over the embolus opening (Fig. 7A, B, D, E); embolus distal margin slightly curved, with a wide and flat opening (Fig. 7A–F).

**Female** (XUX–2011–095; Fig. 6D). Carapace dark yellow with several hairs on the margin; opisthosoma light brown; fourth to eighth tergite light brown with some brown flecks, remaining tergites dark brown; sternum narrow; few fine pointed hairs running over ocular area; chelicerae robust with promargin of cheliceral groove with eleven denticles of variable size; legs with sturdy hairs and spines; opisthosoma with 12 tergites, the second to fifth tergite larger than remaining ones and fourth the largest; seven spinnerets. Measurements: BL 12.30, CL 5.99, CW 5.25, OL 5.67, OW 4.08; ALE > PLE > PME > AME; palp 9.02 (2.91 + 1.05 + 2.18 + 2.88), leg I 11.03 (3.50 + 1.33 + 2.44 + 2.16 + 1.60), leg II 11.11 (3.48 + 1.48 + 2.11 + 2.24 + 1.80), leg III 11.62 (3.22 + 1.41 + 2.17 + 2.96 + 1.86), leg IV 15.92 (4.46 + 1.79 + 2.83 + 4.42 + 2.42).

***Female genitalia*.** The middle pair of the receptacular clusters along the two peaks of the anterior margin of the bursa copulatrix, with obvious genital stalks, the middle receptacular clusters slightly larger or smaller than the lateral ones; the lateral receptacular clusters with obscure genital stalks, situated slightly dorsolaterally, close to the base of the middle genital stalks (Figs 8A–P, 9A–H); the posterior margin of the genital area W-shaped or slightly incurved at the center (Figs 8A–P, 9A–H).

#### Variation.

Males and females vary in body size. The range of measurements in males as follows (*N* = 2): BL 9.67–9.82, CL 4.58–4.91, CW 4.33–4.54, OL 4.64–4.73, OW 3.30–3.33. Females (*N* =20): BL 10.77–14.39, CL 4.68–6.69, CW 4.01–5.92, OL 5.30–7.78, OW 3.52–5.92. In addition, female genitalia show intraspecific variation: the membrane outside the middle genital stalks thick and obvious (Figs 8A–C, 8E–G, L, P, 9B, D, F, H), or thin and obscure (Fig. 8J, K, N, O); the posterior margin of the genital area obviously incurved at the center (Fig. 8A–H, J, N), or obscurely incurved at the center (Figs 8I, K, M, O, 9A, C, D, E, G, H); the middle genital stalks inclined to each other (Figs 8B, D, J, 9A, B), or the short middle genital stalks parallel to each other (Figs 8C, I, L, 9C, D), or one middle genital stalk relatively straight and the other one relatively tilted (Fig. 8A, K); the four receptacular clusters similar size (Figs 8A, B, J, K, 9C, D), or the middle ones larger than the lateral ones (Figs 8C, D, I, 9A), or the middle pair smaller than the lateral pair (Figs 8L, 9B).

#### Remarks.

To confirm whether the specimens we collected are *S. goulouensis*, we attempted DNA extraction on the type specimens, unsuccessfully. We could therefore not assess genetic distances between the four type specimens and the 16 specimens that we had collected. In addition, after closely examining and comparing the types with the original descriptions of Yin (2001), we could not ensure which specimen was used as the holotype to describe the species by Yin (2001). Thus, we assigned a unique code to each specimen (i.e., GL–1997–001 to GL–1997–004) and designated GL–1997–001 as the lectotype. The female genital morphology of all four types was also photographed and presented in Figure 9 for future identification and comparison. Nevertheless, we treat all the specimens as conspecific with *S. goulouensis* for the following reasons: (1) Yin (2001) only described the females, but wrongly described and recognized the genital stalks. After closely examining and comparing the female types with the newly collected females, we found that all the females have comparable morphology, despite considerable intraspecific variation, as is typical of female genitalia in other liphistiids (Xu et al. 2017, 2019): four receptacular clusters similarly sized (Figs 8A, B, J, K, 9C, D) or the middle ones slightly larger than lateral ones (Figs 8C, D, I, 9A) or the middle ones smaller than lateral ones (Figs 8L, 9B), and the middle ones with obvious genital stalks, along the two peaks of the anterior margin of the bursa copulatrix, the lateral ones with obscure genital stalks, situated dorsolaterally, close to the base of the middle genital stalks (Figs 8A–P, 9A–H); (2) the intraspecific genetic distance using K2P model among nine newly collected specimens is very small, 0–0.59% (unpublished data); (3) the specimens (males and females) were collected adjacent to the type locality (Gouloufeng) of *S. goulouensis* (Yin 2001).

#### Distribution.

Hunan (Hengyang) Province, China.

#### GenBank accession number.

XUX–2011–110A: MT102211.

## Supplementary Material

XML Treatment for Songthela

XML Treatment for Songthela huangyang

XML Treatment for Songthela xiangnan

XML Treatment for Songthela goulouensis
